# Dy(III) Doped BiOCl Powder with Superior Highly Visible-Light-Driven Photocatalytic Activity for Rhodamine B Photodegradation

**DOI:** 10.3390/nano8090697

**Published:** 2018-09-06

**Authors:** Jun Yang, Taiping Xie, Chenglun Liu, Longjun Xu

**Affiliations:** 1 College of Materials and Chemical Englineering, Chongqing University of Arts and Sciences, Yongchuan 402160, China; bbyangjun@foxmail.com; 2 Chongqing Key Laboratory of Extraordinary Bond Engineering and Advanced Materials Technology (EBEAM), Yangtze Normal University, Chongqing 408100, China; deartaiping@163.com; 3 State Key Laboratory of Coal Mine Disaster Dynamics and Control, Chongqing University, Chongqing 400044, China; 4 College of Chemistry and Chemical Engineering, Chongqing University, Chongqing 401331, China

**Keywords:** Dy^3+^, BiOCl, photocatalyst, RhB photodegradation, doping modification

## Abstract

Dy-doped BiOCl powder photocatalyst was synthesized A one–step coprecipitation method. The incorporation of Dy^3+^ replaced partial Bi^3+^ in BiOCl crystal lattice system. For Rhodamine B (RhB) under visible light irradiation, 2% Dy doped BiOCl possessed highly efficient photocatalytic activity and photodegradation efficiency. The photodegradation ratio of RhB could reach 97.3% after only 30 min of photocatalytic reaction; this was more than relative investigations have reported in the last two years. The main reason was that the 4f electron shell of Dy in the BiOCl crystal lattice system can generate a special electronic shell structure that facilitated the transfer of electron from valance band to conduction band and separation of the photoinduced charge carrier. Apart from material preparation, this research is expected to provide important references for RhB photodegradation in practical applications.

## 1. Introduction

Photocatalytic technology using visible light irradiation is an environmentally-friendly approach towards environmental pollutant treatment. It has attracted considerable attention due to inexhaustible visible light from solar light energy. Over the past few decades, some visible-light-driven photocatalysts were engineered and fabricated for the photodegradation of organic wastewater. For example, Fe^(0)^ doped g-C_3_N_4_/MoS_2_, fluorinated Bi_2_WO_6_, and TiO_2_ with interface defects [1,2,3] were synthesized, specifically aiming at Rhodamine B (RhB) photodegradation. However, the corresponding photocatalytic reactions were very slow and took several hours to degrade less than 98% RhB, thus impeding their practical application due to the high time costs. If the used light source was simulated visible light, and not solar light, the long times of the photocatalytic reaction would increase energy consumption and costs. Therefore, the challenges we face are how to enhance photocatalytic reaction kinetics, to shorten reaction time, and to boost photocatalytic efficiency under identical visible light irradiation.

Bi-based photocatalysts are important visible-light-responsive photocatalysts and have recently attracted increasing attention. Considering the stability of Bi^3+^, Bi^3+^-containing compounds, such as Bi_2_O_3_, BiVO_4_, Bi_2_WO_6_, BiPO_4_, BiFeO_3_, and BiOX (X = Cl, Br, I) [4], were synthesized for photocatalytic reactions. Most of these compounds, especially BiOX (X = Cl, Br, I), possessed a layered structure and a plate-like appearance that could produce an internal electric field [5,6], which facilitated the migration of photoinduced carriers to some extent. Among these Bi^3+^-based photocatalysts, studies regarding the structure and properties of BiOCl were found. In fact, the photogenerated electrons and holes of BiOCl have not been easily exploited and utilized under visible light irradiation [4,7].

Foreign ion doping has been widely adopted to increase the visible light absorption for single phase photocatalysts, because this process can generate a doping level between the conduction band (CB) and valence band (VB) [8]. Consequently, the energy required to excite electrons is decreased, and the light response of semiconductors is enhanced. Doping modification also steered the charges migrating in a special manner for semiconductors, thus leading to an augmented transfer efficiency of carriers. Hence, doping modifications were commonly employed to boost the photocatalytic activity of Bi-based semiconductors.

Dy-doped ZnO nanoparticles using a photocatalyst were also investigated [9,10,11]. This rare earth metal (Dy) was used as an efficient dopant into the interstitial sites of the ZnO crystal structure. It has been found that rare-earth-metal-doping modification could reduce the electron–hole pair recombination, which is the precondition for efficient photocatalytic applications. Dy could also be incorporated in the crystal lattice of ZnO and thus form ZnO nanoparticles, which could be tuned for optical, morphological and photocatalytic properties. Dy^3+^ ions are well known as an activated dopant for different inorganic crystal lattices, producing visible light by appropriately adjusting yellow and blue emissions.

To the best of our knowledge, no studies have investigated the application of in situ synthesized Dy-doped BiOCl powder photocatalyst for RhB photodegradation in aqueous environments. The as-synthesized Dy-doped BiOCl possessed good photocatalytic activity for the removal of RhB. A total of 97.3% RhB could be degraded in only 30 min of photocatalytic reaction under visible light irradiation.

## 2. Experimental Section

All reagents were of analytical grade purity and were used directly without further purification. Deionized water was used in all experimental processes.

### 2.1. Materials Syntheses

One gram of polyvinylpyrrolidone K30 (PVP K30, Aladdin, Shanghai, China) was completely dissolved in 100 mL distilled water via agitation to form a homogeneous solution that was divided into two parts. A total of 5 mmol Bi (NO_3_)_3_·5H_2_O (Sigma-Aldrich, Hongkong, China) and a proper mol% amount of Dy (NO_3_)_3_·6H_2_O (0.5%, 1%, 1.5%, 2%, 2.5%) (Sigma-Aldrich) were then completely dissolved in the above homogeneous solution by stirring for 30 min. A total of 5 mmol KCl (JZ Chemical, Taoyuan, Taiwan) was also dissolved in the above homogeneous solution by stirring. Then, KCl solution was slowly added into Bi (NO_3_)_3_·5H_2_O and Dy (NO_3_)_3_·6H_2_O mixed suspension under magnetic stirring conditions, and then continuously stirred for 4 h. The mixed solution was filtered. The obtained filter cake was washed several times using 300 mL deionized water and 150 mL absolute ethyl alcohol. The washed filter cake was dried at 80 °C for 10 h to obtain the resultant Dy-doped BiOCl. The pure BiOCl was synthesized using the similar process without adding Dy (NO_3_)_3_·6H_2_O.

### 2.2. Materials Characterization

X-ray diffraction (XRD) measurements were conducted using standard powder diffraction procedures. The samples were smear-mounted on a glass slide and analyzed at a scan rate of 4° (2θ) min^−1^ using monochromatic Cu Kα radiation (MAC Science, MXP18, Tokyo, Japan) at 30 kV and 20 mA. The recorded specific peak intensities and 2θ values were further identified by a computer database system (JCPDS). The chemical compositions of the samples were determined with an X-ray photoelectron spectra (XPS, Physical Electronic ESCA PHI 1600, Chanhassan, MN, USA) at an excitation energy of 1486.6 eV of Al Kα. The C 1s (284.5 eV) signal served as a calibration standard for the Bi and Dy species and their spectra over a wide region. XPS signals of the above species were recorded with a cylindrical mirror analyzer (CMA). The Raman scattering measurements were performed on BiDyxOCl powder samples using an INVIA Raman microprobe (Renishaw Instruments, Wotton-under-Edge, England). The microprobe had an excitation source (488 nm) that was well equipped with a Peltier cooled charge coupled device detector. The morphology, microstructure, and particle size of the as-prepared samples were characterized by field–emission scanning electron microscopy (FE–SEM) (Hitachi, S–4700 Type II, Tokyo, Japan) with a resolution of 0.1 nm and using a high-resolution transmission electron microscopy (HR–TEM) (Hitachi H–7500, Honshū, Japan) at 100 kV, after dispersing the samples on a carbon film supported on a copper grid. The pore volume and surface area of samples were calculated from the nitrogen adsorption–desorption isotherms measured at −196 °C using an ASAP 2010 instrument (micromeritics with surface area deviation of 1%) (ASAP-2010, Micromeritics, Norcross, GA, USA). The optical properties of samples were examined using an ultraviolet–visible diffuse reflectance spectrophotometer (UV-vis DRS, TU1901, Beijing, China).

### 2.3. Photocatalytic Test

One hundred milligrams of powdered photocatalyst were added to 10.0 mg·L^−1^ RhB solution (100.0 mL). The solution was placed in the dark for 1 h, while stirring (500 r·min^−1^) to reach to adsorption–desorption equilibrium (See Figure S1). Single wavelength light-emitting diode (LED) visible light (λ = 470 nm) was used as the visible light source (power = 140 W). At given irradiation time intervals, a series of the reaction solution was sampled and the absorption spectrum was measured.

## 3. Results and Discussion

### 3.1. Phase Analyses

**Figure 1:**
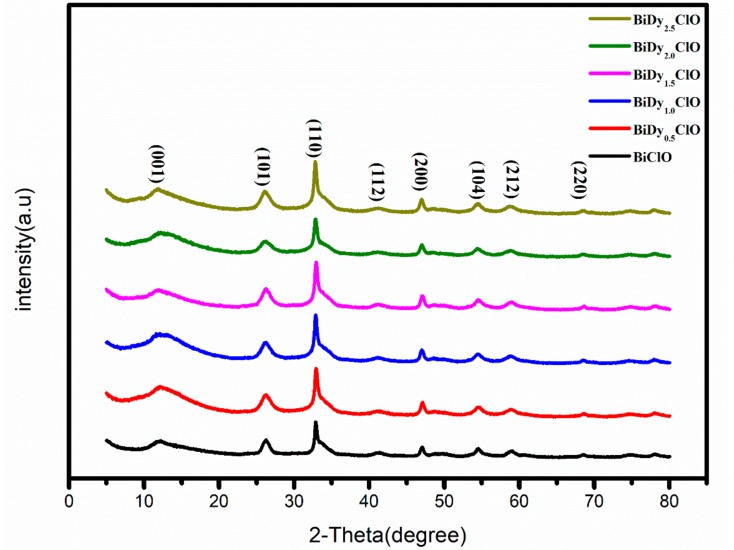
XRD patterns of pure BiOCl and Dy-doped BiOCl.

Figure 1 displays the XRD crystal diffraction patterns of pure BiOCl and Dy-doped BiOCl samples. It can be seen that the diffraction peaks are obviously broadened, indicating that the grain size was smaller, which was due to the size effect of PVP. The smaller grain size contributed to the growth of the final products of smaller particles.

The diffraction peaks of all samples were fully indexed into BiOCl (JCPDS card number: PDF#06-0248) for the tetragonal system [12]. After the introduction of Dy, the diffraction peak of BiOCl had no obvious displacement, which was similar to the investigation of Eu-doped BiOCl. Similar phenomena were observed through investigations of Cu-, Co-, and Fe-doped BiOCl [13,14,15,16]. The Dy_2_O_3_ phase or other impurity peaks were not observed. This was indicated that Dy^3+^ substituted for Bi^3+^ in the BiOCl crystal lattice. The grain sizes of pure BiOCl and (0.5%, 1%, 1.5%, 2% and 2.5%) Dy-doped BiOCl were 9.9, 8.5, 8.3, 7.8, 7.8, and 7.7 nm, determined via calculation using the Scherrer Equation. It can be seen that, with an increase in the Dy doping amount, the grain size of BiOCl gradually decreased, which was due to the distortion of the crystal cell structure caused by the larger ionic radius of the Bi^3+^ ion (r_Bi_^3+^ = 1.17 Å) in the crystal lattice, substituted by Dy^3+^ with a smaller ion radius (r_Dy_^3+^ = 0.91 Å).

**Figure 2:**
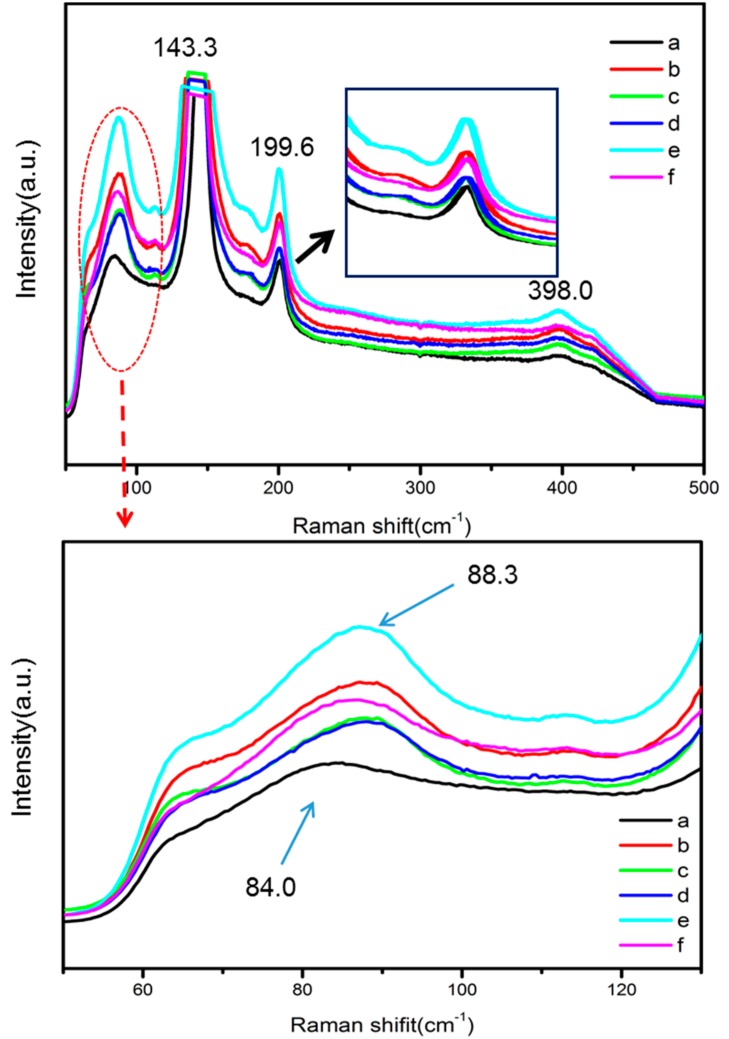
The Raman spectra of (**a**) BiClO; (**b**) BiDy_0.5_ClO; (**c**) Bi0Dy_1.0_ClO; (**d**) BiDy_1.5_ClO; (**e**) BiDy_2.0_ClO; and (**f**) BiDy_2.5_ClO.

The Raman spectra of pure BiOCl and Dy doped BiOCl samples are depicted in Figure 2. The intensity of the symmetric vibration peaks in the Raman spectrum was stronger than that of the asymmetric vibration peaks. The peak at 143.3 cm^−1^ in the spectrum was assignable to symmetrical stretching vibration of the Bi-Cl bond. The peak at 199.6 cm^−1^ was ascribable to the symmetric expansion vibration of the Bi-Cl bond [17]. In addition, the weak and wide peak at 398.0 cm^−1^ was attributable to the oxygen atom vibration peak in the BiOCl system. The asymmetric stretching vibration peak of the Bi-Cl bond should appear at 60.0 cm^−1^, which was not detected here, because the peak intensity of the asymmetric vibration was too weak. Meanwhile, the Raman peaks of BiOCl at 84.0 cm^−1^ and the peaks of BiDy_2.0_OCl at 88.3 cm^−1^ were observed, which was due to the crystal lattice distortion caused by Dy doping into the BiOCl crystal lattice. With the increase in Dy doping amount, the Raman peak at 199.6 cm^−1^ gradually moved to a low wave number, which resulted from grain size reduction with the increase in the Dy doping amount.

**Figure 3:**
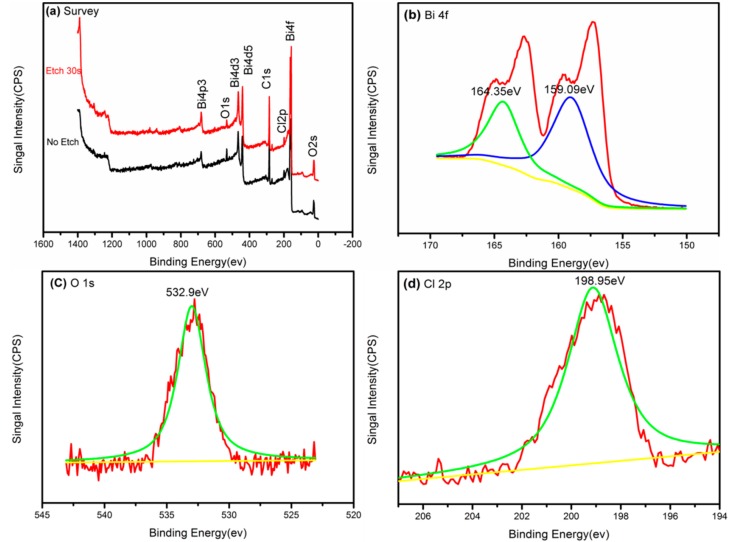
XPS spectra of 2% Dy doped BiOCl (**a**) and the corresponding high-resolution XPS spectra of Bi (**b**); O (**c**); Cl (**d**).

The surface composition and chemical state of a 2% Dy-doped BiOCl sample was analyzed by X-ray photoelectron spectroscopy (XPS), as shown in Figure 3. The Dy element was not detected via common or etch methods, which was attributed to the incorporation of Dy entering into the crystal lattice of BiOCl. Figure 3a shows the XPS full spectra of the original sample and the sample with 30 s of denudation. Only Bi, O, Cl and C were found in the full XPS spectrum of the original sample. The Dy element still could not be detected after etching sample for 30 s. It can be further confirmed that the introduction of Dy^3+^ replaced Bi^3+^ in the BiOCl crystal lattice system. 

Figure 3b–d shows the high-resolution spectra of the etching sample. The peaks at 159.09 eV and 164.35 eV could be ascribable to Bi 4f_7/2_ and Bi 4f_5/2_ spin-orbital Bi^3+^ in BiOCl [18]. The O1s peak at 532.9 eV was from the oxygen atom of the Bi-O bond. The peak of the Cl 2p photoelectron peak appeared at 198.95 eV, corresponding to Cl^−^ in BiOCl.

**Figure 4:**
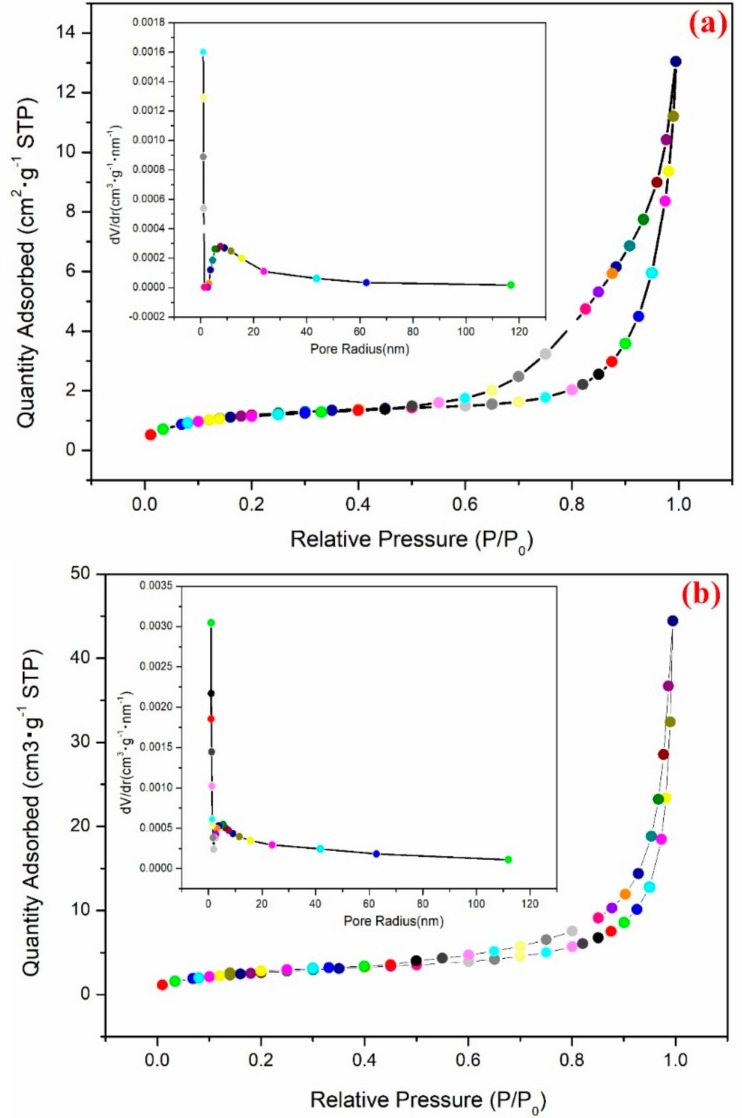
The adsorption–desorption isotherms and the pore size distribution curves (inset). (**a**) BiOCl and (**b**) BiDy_2.0_OCl.

The adsorption–desorption isotherms and the pore size distribution curves (See inset) for pure BiOCl and BiDy_2.0_OCl are shown in Figure 4. The most probable pore-size distributions were 2.76 nm and 2.30 nm for pure BiOCl and BiDy_2.0_OCl, respectively. The introduction of Dy increased the number of micropore and mesopores, which can be confirmed via the adsorption–desorption isotherms. The BET surface areas for BiOCl and BiDy_2.0_OCl were 4.15 m^2^·g^−^^1^ and 9.45 m^2^·g^−^^1^, respectively. Incorporation of Dy could increase the surface area. The smaller ionic radii Dy substituted for Bi could reduce the grain size of BiOCl and further increase its special surface area. The larger the surface area, the more surface-active sites for a catalyst.

### 3.2. Micromorphology Analyses

**Figure 5:**
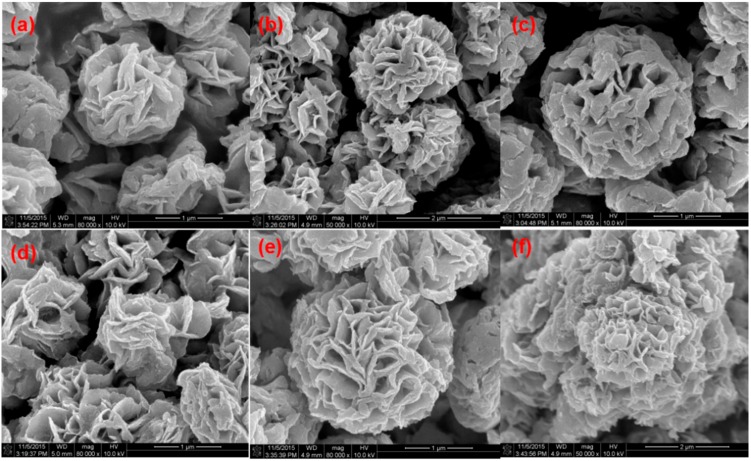
SEM images of BiOCl (**a**) and (0.5%, 1%, 1.5%, 2% and 2.5%) Dy-doped BiOCl (**b**–**f**).

An SEM image of pure BiOCl is shown in Figure 5a. The image presents a spherical-flower structure with a size of 1–2 μm. With the increase in Dy doping amount from 0.5 to 2.5%, the surface structure remained similar, which revealed that the incorporation of Dy did not change the surface morphology and crystal structure of BiOCl. This further confirmed that Dy^3+^ entered into the crystal lattice structure of BiOCl.

**Figure 6:**
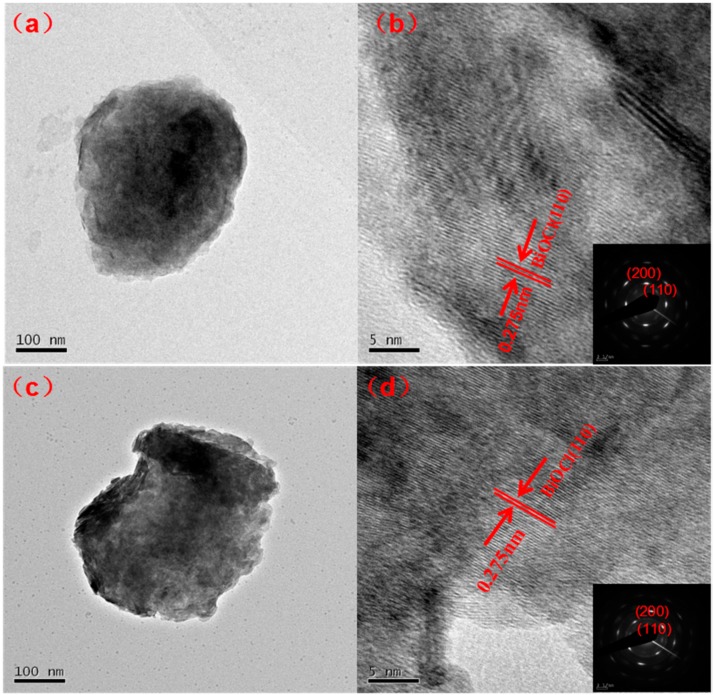
TEM images of BiOCl (**a**), 2% Dy doped BiOCl (**c**) and corresponding HRTEM (**b**,**d**); Inset: SAED pattern of the BiOCl and 2% Dy-doped BiOCl.

Figure 6 shows pure BiOCl and 2% Dy-doped BiOCl transmission electron microscope (TEM) diagrams and the corresponding high transmission electron microscope (HR-TEM, Hitachi H–7500, Honshū, Japan) diagrams, which are illustrations of the selected area electron diffraction (SAED). From Figure 5a,c, it can be seen that the morphologies of pure BiOCl and 2% Dy-doped BiOCl are spherical in structure, which was consistent with the results of the scanning electron microscope test. The results of HRTEM revealed that the crystal lattice stripe was clearly visible and highly consistent, indicating that the crystal structure of the sample was complete and that the crystallinity was good. Further tests indicated that the spacing of the crystal lattice stripe was 0.275 nm [19] (Figure 6b,d), which corresponded to the space between the (110) surface of the BiOCl of the tetragonal system, which was also in accordance with the XRD results. Meanwhile, selective electron diffraction of BiOCl and 2% Dy-doped BiOCl was carried out. As shown in Figure 6b,d, clear diffraction points could be observed. The sample belonged to single crystal, corresponding to BiOCl and Dy-doped BiOCl (110) and (200) surfaces, respectively, thus indicating that the samples had good crystallinity.

### 3.3. Optical Properties

**Figure 7:**
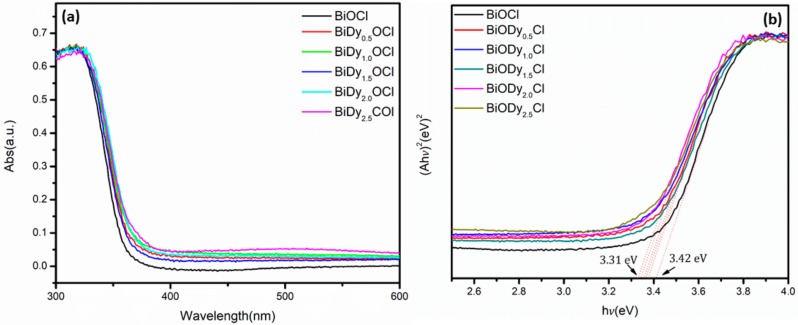
UV–vis diffuses reflectance spectra BiOCl with different Dy contents (**a**) and the corresponding band gap energy (**b**).

Figure 7 displays UV–vis diffuse reflectance spectroscopy results and the corresponding band gap energy diagrams of pure BiOCl and Dy-doped BiOCl.

As can be seen from Figure 7a, with the increase in Dy doping amount, the absorption band of the samples presented a red shift, and the absorption capacity of light increased gradually. Through calculation of the band gap width (Figure 7b), it was found that, when the doping amount was 2.5%, the band gap energy decreased to 3.31 eV from 3.42 eV for pure BiOCl. There was no change in the color of the sample with the introduction of Dy, which implied that Dy showed a change in the electronic structure of the BiOCl, was altered when Dy^3+^ entered the BiOCl crystal lattice and replaced Bi^3+^.

The effect of Mn doping on the electronic structure of BiOCl was thoroughly studied using the first principle [20]. It was found that Mn doping could make the whole energy level of BiOCl move to a low energy level. The incorporation of Mn was in the middle of the band gap and the bottom of conduction band of the BiOCl system, which produced a new impurity level, further reducing the width of the band gap.

Rare earth metal elements possess unique optical properties because of their unique electronic structures. The main reason for this is the existence of the 4f electron shell [21].

The electronic shell structure of rare earth metal elements could be expressed as: 4f^N^5s^2^5p^6^. The electron at 4f shell was not the outermost electron, but indeed the photoactive electrons were at the 4f layer [21]. Therefore, the doping of rare earth metal ions would affect their electronic structure and change their optical properties. Here, the reduction in band gap energy was attributed to the introduction of Dy^3+^ ions which could generate a new electron energy level in the middle of the band gap of BiOCl.

**Figure 8:**
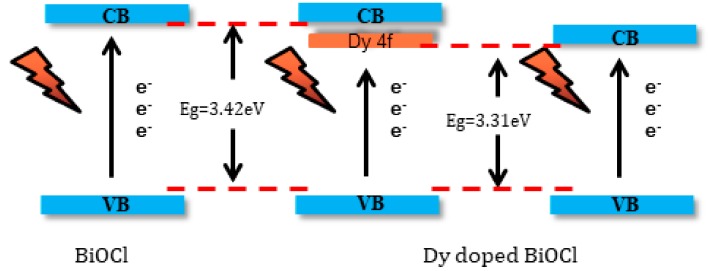
The schematic of reduction in the band gap of BiOCl.

The conduction band of BiOCl was mainly composed of Bi 6p, and the valence band was mainly composed of O 2p, Cl 3p and a small amount of Bi 6s [22]. Electrons were passed from O 2p and Cl 3p to the 4f electronic shell of Dy instead of being directly transmitted to Bi 6p, which can be seen in Figure 8. This was a “springboard” between the valence band and the conduction band, which facilitated the easy jump to the conduction band for photoexcited electrons.

### 3.4. Photocatalytic Activity and Corresponding Mechanism

**Figure 9:**
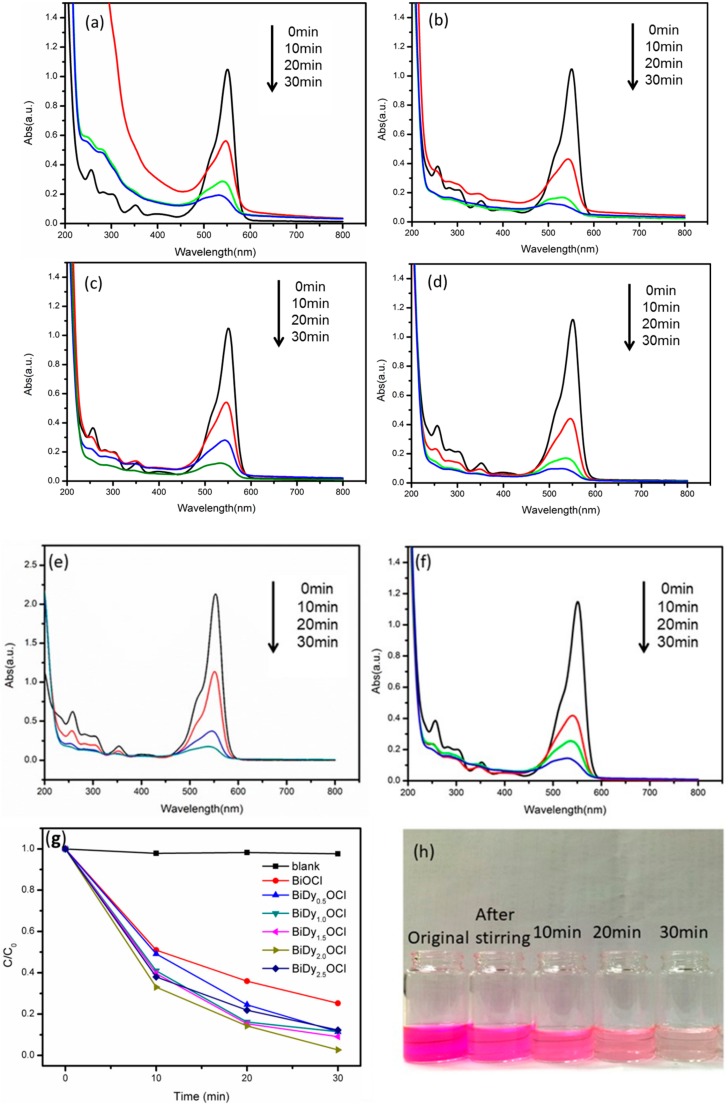
Time-dependent UV-vis absorption spectra of the RhB in the presence of various Dy-doped BiOCl photocatalysts (**a**–**f**) and the corresponding degradation ratio of RhB (**g**). The color change of RhB using 2% Dy doped BiOCl in photodegradation process (**h**).

In order to find the optimal Dy doping amount, the photocatalytic performances of pure BiOCl and Dy-doped BiOCl (0.5%, 1%, 1.5%, 2% and 2.5%) samples were investigated via RhB photodegradation under visible light irradiation. This was also the original intention of this modification study for BiOCl. Here, a single wavelength LED light source was used as simulation visible light due to its low light intensity and securing wavelength. The experimental results are shown in Figure 9.

**Figure 10:**
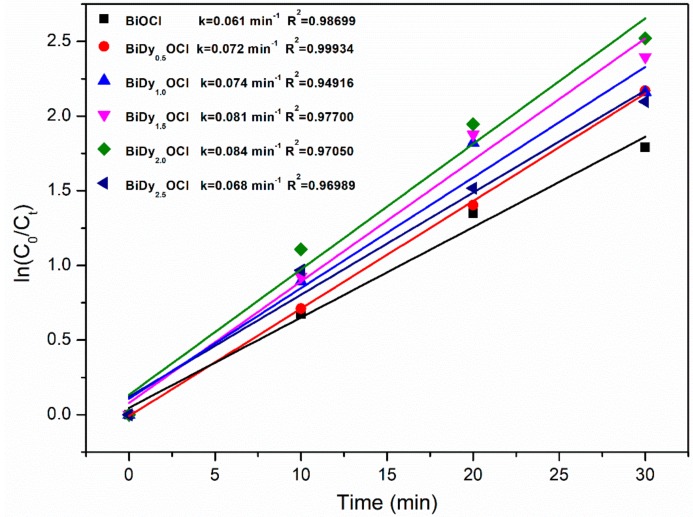
Kinetic linear fitting curve for RhB degradation using different photocatalyst samples.

Figure 9a–f shows the time-dependent UV-vis absorption spectra of RhB in the presence of the as-prepared samples. Figure 9g,h shows the photocatalytic degradation ratio of RhB versus visible light irradiation time and the color change of RhB using 2% Dy-doped BiOCl as a photocatalyst. It was seen that the 2% Dy-doped BiOCl possessed the best photocatalytic activity under identical visible light irradiation. Using 2% Dy-doped BiOCl, the RhB photodegradation ratio reached 97.3%, which was 1.3 times more than pure BiOCl under identical light irradiation (see Table 1). With the increase in the Dy doping amount from 0.5 to 1.5%, photocatalytic efficiency increased tremendously. However, when the Dy doping amount was 2.5%, the RhB photodegradation ratio decreased, which indicated that excessive Dy doping was detrimental to the enhancement of the photocatalytic activity of BiOCl. In addition, the reaction rate constants were determined from the RhB degradation kinetic curves (Figure 10). The reaction rate constant for RhB degradation using 2% Dy-doped BiOCl as photocatalyst was greatest, ca. 0.084 min^−1^, which was more than 1.3 times that of pure BiOCl.

It is worth mentioning that the photocatalytic activity of 2% Dy-doped BiOCl for RhB photodegradation was outstanding. To the best of our knowledge, the photocatalytic ratio reached 97.3% after only 30 min of photocatalytic reaction, and the efficiency was superior to that of existing literature reports (see Table S1). 

Overall the incorporation of Dy can significantly enhance the photocatalytic activity of BiOCl.

First, the band gap energy of BiOCl decreased with Dy doping. In addition, the introduction of the 4f electron shell of Dy was equivalent to adding a “springboard” between the valance band and the covalent band of BiOCl, so that the valence band electrons could be more easily transferred to the conduction band through the 4f electric sublayer of Dy under visible light irradiation. 

Secondly, the electron shell was relatively stable in the semi-full state, and when the Dy^3+^ trapped an electron, the 4f electron shell in the half-full electron state was destroyed, and the stability was reduced, and the electrons were released to the stable state. The released electrons reacted with the oxygen adsorbed on the surface of the sample to produce a photocatalytic active substance. In fact, a dye sensitized electron was possibly captured by Dy^3+^, so the 4f electron shell could be used either as an electronic conductor or as a collection of electrons. The two processes could facilitate the separation of photoinduced electron–hole pairs and further improve photocatalytic activity. 

Finally, when the Dy doping amount was 2.5%, the photocatalytic activity decreased. The incorporation of Dy provided a “springboard” for the low energy light activation electron transition, but excessive doping amounts would give rise to scattered distributions for the produced impurity energy, which would generate a recombination center for the electrons and holes.

In fact, environmental pollution resulting from dye wastewater is becoming more and more serious, which prompted humanity to realize the importance of green chemistry (environmentally-friendly chemistry) [23] that advocates for existing chemistry technologies and methods to be used to reduced or stop hazards to human health, community safety and the ecological environment. At present, some reports [24,25,26] have opened new frontiers in the field of catalysis. A super membrane technology (membrane-grafted catalyst) could reduce the emissions of reaction by-products and the recovery of residual solvents, which would be a good research direction. 

## 4. Conclusions

Dy-doped BiOCl powder photocatalyst was synthesized using a one-step coprecipitation method. The incorporation of Dy^3+^ was successfully substituted for a part of Bi^3+^ in the BiOCl crystal lattice system. Two-percent Dy-doped BiOCl possessed the best photocatalytic activity and photodegradation efficiency for RhB under visible light irradiation. The photodegradation ratio of RhB reached 97.3% within 30 min of photocatalytic reaction under visible light irradiation. The main reason for this is that the 4f electron shell of Dy in the BiOCl crystal lattice system could generate a special electronic shell structure that facilitated the transfer of electrons from the valance band to the conduction band and also the separation of the photoinduced charge carrier. This work hopes to provide an important reference for RhB photodegradation in practical applications using this photocatalyst. 

## Figures and Tables

**Table 1:** The photodegradation ratio for RhB using BiOCl and Dy doped BiOCl samples. A total of 100.0 mg of powder photocatalyst was put into 10.0 mg·L^−^^1^ of RhB solution (100.0 mL).

Time (min)	BiOCl	BiDy_0.05_OCl	BiDy_0.1_OCl	BiDy_0.15_OCl	BiDy_0.2_OCl	BiDy_0.25_OCl
10	49.0%	51.0%	59.0%	60.3%	67.0%	62.0%
20	64.0%	74.5%	83.8%	84.7%	85.7%	78.1%
30	74.8%	88.6%	88.5%	90.9%	97.3%	87.7%

## Supplementary Materials

The following are available online at http://www.mdpi.com/2079-4991/8/9/697/s1, Figure S1: The degradation rate of RhB with BiDy_2.0_OCl in dark, Table S1: Comparison of photodegradation ratios using different photocatalysts under visible light irradiation (reported in the last two years).
