# Supplementary material for: Dy(III) Doped BiOCl Powder with Superior Highly Visible-Light-Driven Photocatalytic Activity for Rhodamine B Photodegradation
Source: Nanomaterials (Basel). 2018 Sep 6;8(9):697. doi: 10.3390/nano8090697 (PMC6164470; doi:10.3390/nano8090697)
Supplement: Supplementary file 1 [file nanomaterials-08-00697-s001.pdf]

Supplementary Materials

Dy(III) Doped BiOCl Powder with superior highly Visible-Light-Driven Photocatalytic Activity for Rhodamine B Photodegradation

Jun Yang [1,†], Taiping Xie [2,3,†], Chenglun Liu [3,4,*] and Longjun Xu [3,*]

[1] College of Materials and Chemical Englineering, Chongqing University of Arts and Sciences, Yongchuan 402160, China; bbyangjun@foxmail.com
[2] Chongqing Key Laboratory of Extraordinary Bond Engineering and Advanced Materials Technology (EBEAM), Yangtze Normal University, Chongqing 408100, China
[3] State Key Laboratory of Coal Mine Disaster Dynamics and Control, Chongqing University, Chongqing 400044, China; xlclj@cqu.edu.cn (C.L.)
[4] College of Chemistry and Chemical Engineering, Chongqing University, Chongqing 401331, China
* Correspondence: xlclj@cqu.edu.cn (C.L.) and xulj@xqu.edu.cn (L.X.)
† These authors contributed equally to this work.

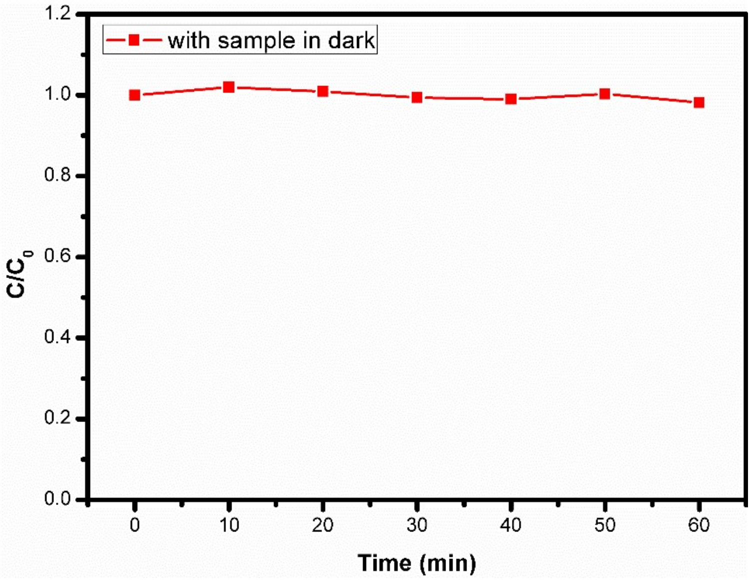

Figure S1. The degradation ratio of RhB with BiDy2.0OCl in dark.

As shown in Figure S1, after reaching to the adsorption – desorption equilibrium via stirring for 1h in the dark, the solution was kept stirring for 1 h, and the absorbance was measured every 10 mins. The results showed that the absorbance of RhB did not change

significantly with the increase of time after reaching adsorption–desorption equilibrium, indicating that RhB would not degrade in the presence of a photocatalyst without light irradiation.

Table S1. Comparison of photodegradation ratio using different photocatalysts under visible light irradiation (reported in the last two years).

Photocatalysts	Photodegradation Ratio	Photodegradation Reaction Time (min)	Refs.
Dy-doped BiOCl	97.30%	30	This work
Ag_3PO_4 nanoparticles	80.50%	45	[1]
$g\text{-}C_3N_4$ with sacrificial KIT-6 template	100%	50	[2]
P-doped $g\text{-}C_3N_4$	100%	50	[3]
Copper fiber@ZnO/CdS	90%	60	[4]
Square-sharped BiOCl nanosheets	98%	60	[5]
2D MoS_2/Red phosphorus heterojunction	97.50%	80	[6]
Zero Valent $Bi^{(0)}$ incorporated bismuth terephthalate	97%	80	[7]
CdS/Ag/a-TiO_2	82%	80	[8]
MIL-88A(Fe)/grapheme oxide composite	100%	100	[9]
Zero Valent $Fe^{(0)}$ doped $g\text{-}C_3N_4/MoS_2$	98.20%	150	[10]
Hexagonal/monoclinic-WO_3	91%	180	[11]
Fluorinated Bi_2WO_6	98%	210	[12]
TiO_2 with interface defects	75%	300	[13]

It is worth mentioning that the photocatalytic activity of 2% Dy-doped BiOCl for Rhodamine B (RhB) photodegradation was outstanding. To the best of our knowledge, the photodegradation ratio of

RhB could reach 97.3% after only 30 min of photocatalytic reaction under visible light irradiation. The photocatalytic efficiency was superior to that in existing literature reports.

References:

[1]B.B. Xu, X.J. Wang, C.F. Zhu, X. Ran, T.F. Li, L.J. Guo, Probing the inhomogeneity and intermediates in the photosensitized degradation of rhodamine B by Ag_3PO_4 nanoparticles from an ensemble to a single molecule approach. RSC Adv., 7(2017)40896–40904.

[2]L. Luo, A.F. Zhang, M. J.Janik, K.Y. Li, C.S. Song, X.W. Guo, Facile fabrication of ordered mesoporous graphitic carbon nitride for RhB photocatalytic degradation. Appl. Surf. Sci., 396(2017)78-84.

[3]J.J. Feng, D.K. Zhang, H.P. Zhou, M.Y. Pi, X.D. Wang, S.J. Chen, Coupling P nanostructures with P-doped g-C_3N_4 as efficient visible light photocatalysts for H2 evolution and RhB degradation. ACS Sustainable Chem. Eng., 6(2018)6342–6349.

[4] Z.J. Yu, M.R. Kumar, Y. Chu, H.X. Hao, Q.Y. Wu, H.D. Xie, Photocatalytic decomposition of RhB by newly designed and highly effective CF@ZnO/CdS hierarchical heterostructures. ACS Sustainable Chem. Eng., 6(2018)155–164.

[5] Y.J. Cai, D.Y. Li, J.Y. Sun, M.D. Chen, Y.R. Li, Z.W. Zou, H. Zhang, H.M. Xu, D.S. Xia, Synthesis of BiOCl nanosheets with oxygen vacancies for the improved photocatalytic properties. Appl. Surf. Sci., 439(2018)697-704.

[6] X. Bai, J. Wan, J. Jia, X.Y. Hu, Y.D. He, C.L. He, E.Z. Liu, J. Fan, Simultaneous photocatalytic removal of Cr(VI) and RhB over 2D MoS_2/Red phosphorus heterostructure under visible light irradiation. Mater. Lett., 222 (2018) 187–191.

[7] X.Y. Zhao, J.P. Zhong, J.C. Hu, L.M. Wu, X. Chen, Bismuth terephthalate induced Bi(0) for enhanced RhB photodegradation and 4nitrophenol reduction. J. Phys. Chem. Solids, 111 (2017) 431-438.

[8] H.J. Liang, S.N. Liu, H.C. Zhang, X.B. Wang, J.J. Wang, New insight into the selective photocatalytic oxidation of RhB through a strategy of modulating radical generation. RSC Adv., 8(2018)13625–13634.

[9] N. Liu, W.Y. Huang, X.D. Zhang, L. Tang, L. Wang, Y.X. Wang, M.H. Wu, Ultrathin graphene oxide encapsulated in uniform MIL-88A(Fe) for enhanced visible light-driven photodegradation of RhB. Appl. Catal., B, 221(2018)119–128.

[10]X. Wang, M.Z. Hong, F.W. Zhang, Z.Y. Zhuang, Y. Yu, Recyclable Nanoscale Zero Valent Iron Doped g-C_3N_4/MoS_2 for Efficient Photocatalytic of RhB and Cr(VI) Driven by Visible Light. ACS Sustainable Chem. Eng., 4(2016)4055–4063.

[11] Y. Lu, J. Zhang, F.F. Wang, X.B. Chen, Z.C. Feng, C. Li, K_2SO_4-Assisted hexagonal/monoclinic WO_3 phase junction for efficient photocatalytic degradation of RhB. ACS Appl. Energy Mater., 1(2018) 2067–2077.

[12]H.B. Fu, S.C. Zhang, T.G. Xu, Y.F. Zhu, J.M. Chen, Photocatalytic Degradation of RhB by Fluorinated Bi_2WO_6 and Distributions of the Intermediate Products. Environ. Sci. Technol., 42(2008)2085–2091.

[13]J.D. Zhuang, W.X. Dai, Q.F. Tian, Z.H. Li, L.Y. Xie, J.X. Wang, P. Liu, Photocatalytic Degradation of RhB over TiO_2 Bilayer Films: Effect of Defects and Their Location. Langmuir, 26(2010)9686–9694.
